# Exploring How System Dimensions and Periodic Boundary Conditions Influence the Molecular Dynamics Simulation of A_6_H Peptide Self-Assembly Nanostructures

**DOI:** 10.1021/acs.jpcb.4c03043

**Published:** 2024-07-09

**Authors:** Karinna Mendanha, Guilherme Colherinhas

**Affiliations:** Instituto de Física, Universidade Federal de Goiás, Goiânia, GO 74690-900, Brazil

## Abstract

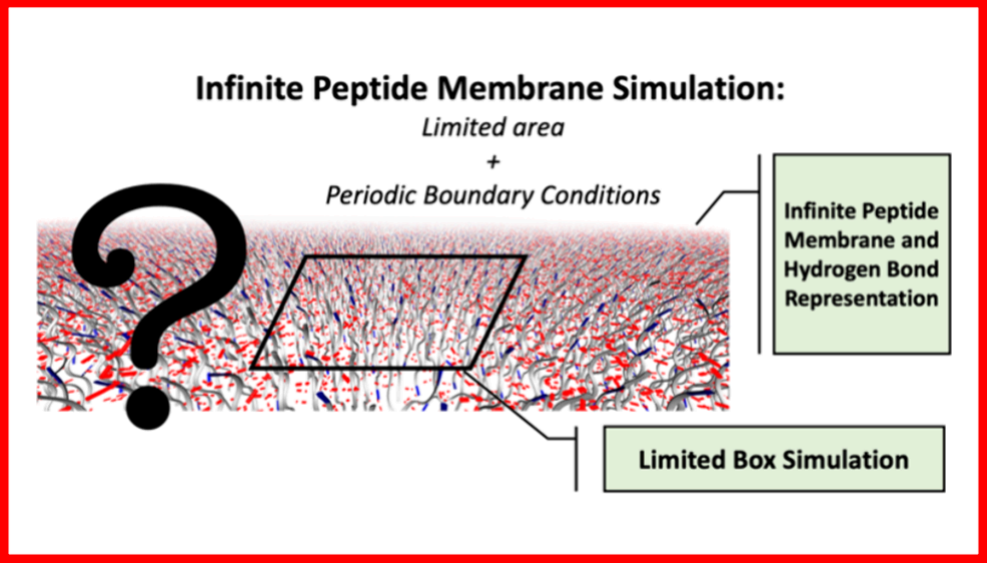

This work presents a study on the effects of periodic boundary conditions (PBC) on the energetic/structural properties and hydrogen bond dynamics (HB) using molecular dynamics (MD) simulations of peptide membranes composed of alanine and histidine. Our results highlight that simulations using small surface areas for the peptide membrane may result in nonconvergent values for membrane properties, which are only observed in regions simulated at a certain distance from the PBCs. Specifically, regarding hydrogen bonds, a property pervasive in peptide membranes, our findings indicate a significant increase in the lifetime of these interactions, reaching values ∼19% higher when observed in structures free from PBCs. For peptide mobility in these nanomembranes, our results compare regions simulated directly under the influence of PBCs with regions free from these conditions, emphasizing greater mobility of amino acid psi/phi angles in the latter model.

## 1 Introduction

Theoretical and experimental studies have indicated the significant potential of amphiphilic structures in the formation of nanostructured materials.^1−4^ These structures can be incorporated into biologically active materials, making them highly applicable in the field of biomaterials.^5,6^ Recent articles further underscore that amphiphilic structures are capable of giving rise to variousmaterials, including micelles, nanotubes, and nanosheets.^7−15^ Research demonstrates thatdipeptides composed of -histidine have potential as neuromodulators and neuroprotective agents.^16,17^ Additionally, structures composed of alanine, such as A_6_R, exhibit antimicrobial characteristics.^18^ Therefore, the experimental work carried out by Castelletto et al.^19^ investigated the self-assemblyprocess of A_6_H structures, demonstrating that, when mixed with water, they can formnanostructures such as ribbons and sheets. The results of Cryo-TEM experiments conducted on A_6_Hsamples with different concentrations showed the formation of self-organized structures in these samples. The sample with 18% weight of A_6_H exhibited large aggregates resembling ribbons, while the sample with 9% weight of A_6_H formed long sheets that coiled into helical structures.

A theoretical way to study peptide structures with high reliability is through Molecular Dynamics simulations (MD). However, the process of molecular modeling may involve several approximations, and during MD computational simulations, it is possible, in some cases, to identify undesired effects that do not accurately represent the real macroscopic system. Therefore, a very small system, despite being quickly simulated computationally, may indicate inaccurate characteristics during the simulation, while a very large system, capable of assessing important features, may be impractical for the computational simulation process even on high-performance computers. One way to mitigate such effects is by controlling the dimensions of the simulated box through the imposition of periodic boundary conditions (PBC). In this approach, if an atom moves to one end of the box, another atom automatically enters from the opposite direction, maintaining constant the total number of atoms in the system. This strategy also ensures that an infinite system can be simulated from a finite system since, with PBCs, the simulation box is centered and replicated in all directions. This strategy is valid and applied in various areas, for example, the work of Barclay and Zhang^20^ studied the use of PBCs for studying equations of state in systems with a high rate of deformation. However, they showed that, similar to systems using PBCs, the gLE-PBC also has a limitation when there is compression in a specific direction in the system. Other article, such as Hunt,^21^ demonstrate the use of new PBCs to solve problems in simulations of planar extensional flow, and in this case, they conducted studies on simple liquids out of equilibrium using these new PBCs and showed that the technique allows for results that agree with other simulations. Specifically for membranous structures (lipid-based, for example), there are several studies discussing the influence of PBCs on the properties of the simulated membrane, highlighting the influence of the simulated model size.^22−26^ The choice of membrane size to be simulated is of utmost importance for the accuracy and validity of results obtained in MD simulations. In the article by Herce and Garcia,^27^ it was demonstrated that the average area per lipid increases with the size of the simulated unit cell, making it necessary to individually couple each degree of freedom to the thermal bath to correct this finite size effect. In work by Castro-Román et al.^28^ was observed that, in the absence of external stress, the surface tension of a lipid membrane vanishes at equilibrium, but long-wavelength fluctuations are generally suppressed in small simulation areas, impacting the proper modeling of the stress-free state of macroscopic membranes. The article by Klauda et al.^29^ highlights that the lateral diffusion constant of lipids varies significantly with the size of the simulated system, showing a dramatic finite size effect that affects the diffusion correlation between lipids. Therefore, the choice of simulated membrane size is crucial to avoid artifacts and ensure that the observed dynamic and structural properties are representative of real systems, justifying the need for larger-scale simulations and refinements in electrostatic cutoff methods.

Therefore, despite relatively success, the use of PBCs can be problematic in describing the behavior of fiber/membrane-like structures. For fibers cases, the use of a short fiber may obscure interpretations of ripple and twist effects, as the fiber’s ends are confined within the same degree of freedom. This effect was demonstrated by de Andrade et al.^4,30^ For membranes, restricting the simulation area of the membrane creates constraints at the membrane edges that may hinder the converged analysis of a structural or energetic property. However, choosing the dimensions of the simulated structure and managing computational costs are challenging balancing acts. In this work, our objective is to explicitly evaluate the effects of increasing the simulation area of a peptide membrane composed of A_6_H, considering a direct comparison between a small and large surface system. The A_6_H system was chosen due to its composition of a reasonably short peptide that forms membranes with a high alignment of peptide β-sheets, and it has results discussed and compared both theoretically and experimentally.^31^ Our focus is not on a discussion of the membrane properties’ results per se but rather on how they may differ when considering the simulation of a membrane with an area nine times larger than the conventional size. Thus, the impact of the constraints imposed by PBCs can be disregarded when statistics are conducted over a portion of the macro system. Thus, despite the challenge and significant computational effort, the present work strives to assess the effects of increasing the simulation area of a peptide membrane (such as A_6_H), aiming to provide a valuable contribution to understanding how the system size influences the results of systematically and delicately conducted classical simulations. This endeavor offers a comprehensive perspective on the implications of PBCs in nanomembrane simulations, along with an estimate of the errors that may arise when such an approach is applied.

## 2 Methodology

**Figure 1:**
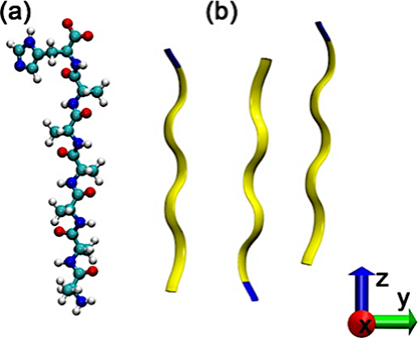
(a) Monomer represented by atoms and ribbon pictures. (b) Dimer of two A_6_H peptides formed by alanine (A) and histidine (H). Yellow represents alanine, and blue represents histidine amino acids.

For the purpose of this work, a peptide was constructed using the Pymol program^32^ based on a sequence of the amino acids: six alanine (A) and one histidine (H) molecule with a β-sheet structure, with *zwitterionic* termination −NH_3_^1+^ and −COO^1–^ mapped by the CHARMM36 force field.^33^ Thus, using GROMACS tools,^34^ a dimer was constructed using two A_6_H monomers, one inverted relative to the other, as shown in Figure 1. This dimer was then replicated 10:10 times on *x-* and *y*-axes, forming the β-sheet nanomembrane structure A_6_H (referred to here as A_6_H-11), favoring hydrogen bonds (HBs) that are crucial for maintaining β-sheet nanostructures.^7,30^ This structure was then solvated in water (modeled with TIP3P^35−37^), forming two layers of water molecules (about 3 nm each), positioned in contact with each of the membrane surfaces.

**Figure 2:**
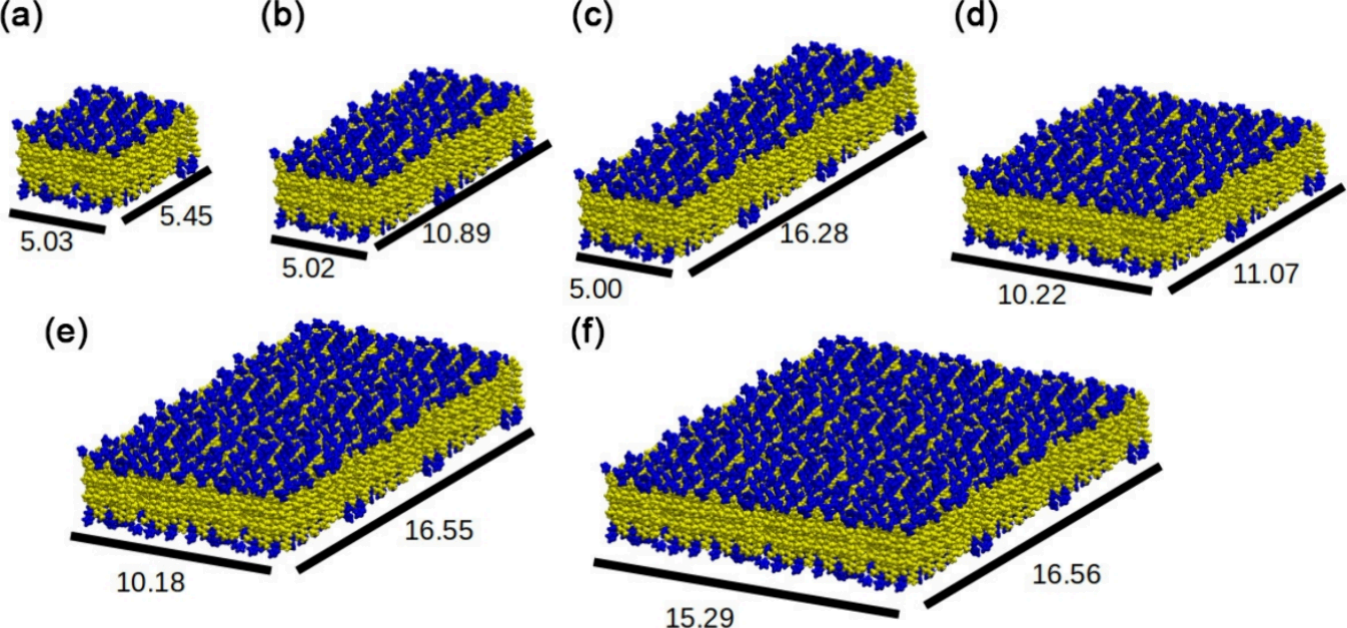
Initial peptide nanomembranes structures (*x* and *y* dimension box in nm). (a) A_6_H-11; (b) A_6_H-12; (c) A_6_H-13; (d) A_6_H-22; (e) A_6_H-23; and (f) A_6_H-33. Yellow represents alanine, and blue represents histidine.

The solvated A_6_H-11 nanomembrane model was subjected to a sequential MD simulation, alternating, and every 2 ns, between the NPT and NVT thermodynamic ensembles. This procedure was carried out until thermodynamic equilibrium was reached (about 30 ns of MD simulation). After this initial sequence of MD simulations, new nanomembranes models were constructed based on replicas of the equilibrated A_6_H-11 membrane configuration. The A_6_H-12 model has one replica of along the *x*-axis and two replicas along the *y*-axis. The difference between these two models is that one dimension of A_6_H-11 will not have the same constraints due to PBCs, allowing for greater freedom in the movement of peptides. The A_6_H-13 model has one replica along the *x*-axis and three replicas along the *y*-axis, while the A_6_H-22 model has two replicas along both axes, allowing for increased freedom in organizing the nanomembrane due to the initial structure comprising 25% of the new structure. Similarly, the A_6_H-23 model has two replicas along the *x*-axis and three replicas along the *y*-axis, whereas the A_6_H-33 model features 3 replicas along both axes, forming a box with nine blocks of the initial structure. A direct comparison between the A_6_H-11 structure and center of A_6_H-33 will provide insights into how A_6_H peptide nanomembranes behave in an environment without the constraints imposed by PBCs. Figure 2 provides a detailed illustration of the initial configuration for each of the models simulated (only peptide membranes). Following the membrane construction procedure, all systems underwent solvation in water molecules to enable the application of new PBCs, thereby ensuring system continuity and preventing interactions between them and their replicated versions along the *z*-axis. To accomplish this, a water layer was incorporated above and below the two surfaces of the nanomembrane, thereby finalizing the simulation boxes.

All new models are subjected to a new sequence of MD-NPT and MD-NVT simulations for a new thermodynamic equilibration, this time for 30 ns. Subsequently, and after confirming that all systems are in thermodynamic equilibrium, a new MD-NPT production phase is carried out, where the systems are simulated for 100 ns. From this production phase, we saved the trajectory for statistical analysis of the properties to be presented in this work. In the supporting material, we demonstrate an analysis of the potential energy behavior of the systems as a function of simulation time, highlighting the behavior that proves that they are all in thermodynamic equilibrium during the production stage (Figure S1). Each MD step was performed with a time step of 1 fs, and a total of 10^8^ MD steps were conducted in the production phase of the MD simulation. To calculate the electric potential, the Particle–Mesh Ewald (PME)^38^ method was employed with a cutoff radius of 1.2 nm, and for the van der Waals energies, the Potential-Shift Verlet method was utilized with a cutoff radius of 1.2 nm. In all MD-NPT simulations, a pressure of 1.013 bar was maintained using semi-isotropic Parrinello–Rahman^39^ coupling, with adjustment every 4 ps, and compressibility of 4.5 × 10^–5^ bar^–1^. We emphasize that, in the context of simulations with finite-sized membranes, it is acceptable for the pressure methodology. However, membranes have a finite surface tension, which may hinder comparison with experimental data.^40^ To keep the temperature constant at 300 K, the v-rescale^41^ algorithm was employed every 0.1 ps. For the analysis of hydrogen bonds (HBs), we calculated the average values for the total number of these interactions based on the typical configurational conditions (*r* ≤0.35 nm and Theta(acceptor–hydrogen–donor) ≤30 degrees). Additionally, we also calculated the lifetime of these interactions and the cutoff energy value for the breaking of HBs as described by the theories of Luzar and Chandler^42,43^ and Van Der Spoel et al.,^44^ which combine HB statistics with temporal correlation. The LINCS^45^ algorithm was used to constrain the bond lengths. The images were obtained using the VMD program,^46^ and the analyses were performed using the GROMACS software package, and additionally, the SuAVE program^47^ was also utilized in the study. Table 1 presents numerical details of the simulation boxes. With the same computational resources (8 processors at 3800 MHz), the computational expenditure for each simulation during the production stage of every A_6_H nanomembrane model varied, being 1.7, 2.8, 3.9, 5.9, and 8.4 times the simulation duration for the A_6_H-11 model, correspondingly for the A_6_H-12, A_6_H-13, A_6_H-22, A_6_H-23, and A_6_H-33 models.

**Table 1:** Composition of Simulation Boxes Containing Nanomembranes of All A_6_H Models^a^

parameter	A_6_H-11	A_6_H-12	A_6_H-13	A_6_H-22	A_6_H-23	A_6_H-33
# peptides (*N*)	100	200	300	400	600	900
# waters (Nw)	5922	11,844	17,766	23,688	35,532	53,298
# total atoms (Na)	25,766	51,532	77,298	103,064	154,596	231,894
final average superficial area (*S*)	27.45	54.74	81.56	113.16	168.51	253.17
final average box’s volume (*V*)	244.80	489.59	771.62	979.46	1470.74	2203.48
final average # peptides/area (*N*/*S*)	3.64	3.65	3.67	3.53	3.56	3.55

a Number of peptides in nanomembrane simulated (*N*); number of water molecules in simulation boxes (Nw); number of total atoms in simulation boxes (Na); pos-production average superficial area of peptide membrane (*S*, in nm^2^); pos-production average volume of simulation boxes (*V*, in nm^3^); and pos-production average proportion of number of peptides (*N*) per superficial membrane area (*S*, in # peptide/nm^2^).

## 3 Results and Discussion

Below, we summarize the key findings derived from MD simulations. The structural analysis aims to demonstrate the condensation of the structure following MD production simulation, encompassing an evaluation of membrane thickness to enable direct correlation with experimental results. We investigate Coulomb and Lennard–Jones interaction energies to elucidate peptide–water interactions, evaluating convergence or alterations when comparing systems of different sizes. An approximation of the energy variance between A_6_H-11 and A_6_H-33 models will be provided. Moreover, we will present statistics and dynamics concerning hydrogen bonds, highlighting the significance of examining a diminished configurational space versus a broader one.

### 3.1 Coulomb and van der Waals Interactions Energies

#### 3.1.1 Coulomb Interaction Energy

**Figure 3:**
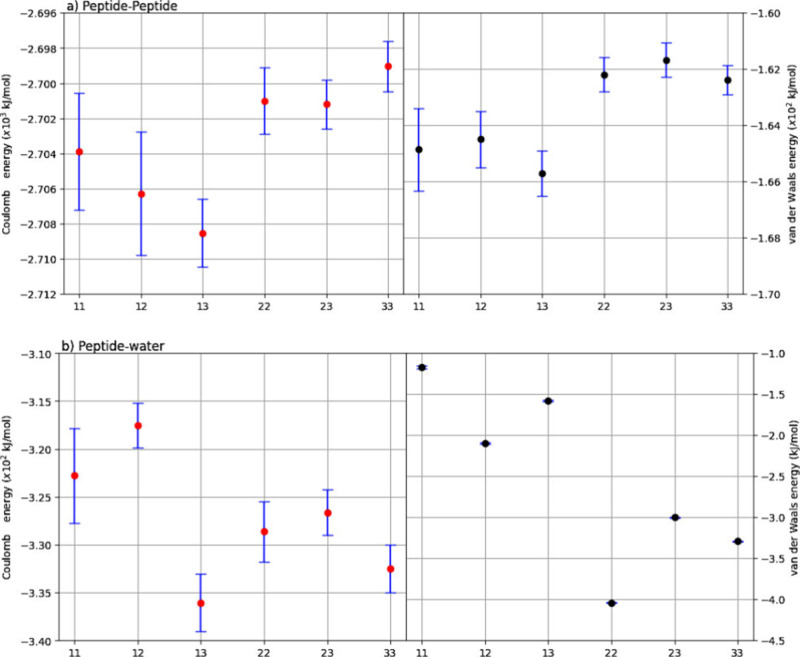
Average Coulombic (red) and van der Waals (black) energy for interactions between (a) peptide–peptide and (b) peptide and water molecules, in kJ/mol (per peptide), for the all A_6_H-XX models.

In this section, we present the Coulombic interactions energy (*E*_C_) between residues, as well as between residues and water molecules, measured in kJ/mol per peptide (*N*) unit in the nanomembrane system. All values for this property (and RMSD) are available in the supporting material (Table S1), and Figure 3 [Figure S2] shows the Coulomb energies between peptides [residues] and between peptides and water [residues and water] molecules for each simulated system. These results indicate that the *E*_C_(Ala-Ala) remains consistent within a variation of merely 0.1% across the studied models, suggesting that the increase in system size does not notably affect this interaction, which primarily resides within the membrane. Regarding *E*_C_(Ala-water), the results reveal a variation ranging from approximately −2.3 to 3.8% when comparing the values obtained for model A_6_H-11 with those of models A_6_H-12 and A_6_H-33. Conversely, the interaction between Ala-His (pertaining to membrane’s surface residues) and between His-His (in the central membrane region) shows a minimal difference, less than 1%, among the models. Despite these subtle differences between residues, the Coulombic interaction between histidine and water molecules may play a crucial role in defining the hydrophilic/hydrophobic characteristics of the membrane surface. In this context, *E*_C_ interactions for Ala-water have demonstrated influence as the membrane area increases, consequently influencing a dependence on *E*_C_(His-water) as the simulated membrane size expands, thereby enhancing the interaction between histidine and water. Notably, when comparing models A_6_H-11 and A_6_H-33, the increase in membrane surface area can lead to an up to 2.5% rise in the *E*_C_ value for histidine–water interaction, and comparing models A_6_H-11 and A_6_H-13, the enlargement of the membrane’s surface area can result in a reduction of up to 5% in the *E*_C_ value.

#### 3.1.2 Van der Waals Interaction Energy

Figure S2 also display the average values for van der Waals (*E*_VDW_) energy interactions among residues and between residues and water molecules. *E*_VDW_(Ala-Ala) average values indicate that model A_6_H-13 exhibits a stronger attractive interaction compared to the other structures. Model A_6_H-12 [A_6_H-13] shows a variation of 0.09% [1.06%] compared to reference model A_6_H-11. Model A_6_H-22 [A_6_H-23] shows a variation of 1.92% [2.14%] and model A_6_H-33 shows a variation of 1.84% when compared to model A_6_H-11. These variations indicate that, energetically, there is no significant change between the values of van der Waals interactions among alanine residues in center of membranes. However, when compared to the values obtained for *E*_C_(Ala-Ala) interactions, they are approximately 13–14 times smaller. For *E*_VDW_(Ala-water), the average values indicate that, with an increase in the membrane simulated surface, the interaction between the hydrophobic region and water molecules becomes attractive. The *E*_VDW_(Ala-His) is close to −20 kJ/mol.N, and *E*_VDW_(His-His) values are close to −9 kJ/mol.N. Finally, for *E*_VDW_(His-water), the A_6_H-13 model shows a higher interaction between the hydrophilic region and solvent than another structure. Comparing these values with model A_6_H-11, we observe variations between 2 and 33%. These *E*_VDW_ values do not change significantly with the increase in membrane surface; however, there are considerable percentage variations between models.

This indicates a clear conclusion that, energetically, the membrane does not exhibit a global modification in the values of *E*_C_ or *E*_VDW_. However, the findings suggest that the interaction energy between residue and water may have distinct interpretations when a larger surface membrane is considered in the simulation process. We emphasize that these results for vdW and Coulomb energies do not invalidate previous results obtained for smaller membrane models but highlight that, energetically, the estimate obtained with smaller models can be satisfactory, depending on the case evaluated. For a better analysis of this impact, below, we will perform a direct comparison of the interaction energy values exclusively for A_6_H-11 and the center of the A_6_H-33 membrane, which represents exactly the same group of peptides as A_6_H-11 but free from PBCs.

#### 3.1.3 Comparing *E*_C_ and *E*_VDW_ for A_6_H-11 with the Center of Model A_6_H-33

At this point, we will examine the results regarding *E*_C_ and *E*_VDW_, comparing the A_6_H-11 model (with a smaller surface area) against a corresponding segment located in the center of the A_6_H-33 model. This comparison aims to assess the effects of the presence of PBCs (on the periphery of the A_6_H-11 model), observing a region of equivalent surface area devoid of PBCs (central area of the A_6_H-33 model), within the A_6_H-type peptide membranes. We emphasize that this comparison can be understood as a metric to evaluate membrane models formed by peptides of this nature. Naturally, the *E*_C_ and *E*_VDW_ values obtained for the A_6_H-11 model cover interactions between molecules located on opposite sides of the simulation box, due to PBCs. These interactions are absent in the values obtained for the central segment of A_6_H-33. Consequently, we also obtained *E*_C_ and *E*_VDW_ results for the region surrounding the central segment of the A_6_H-33 model. This approach allows us to emulate the contribution attributable to PBCs in this model, denoted as *E*_X_ = *E*_Total_ – *E*_Center_ – *E*_Edge_. This *E*_X_ energy specifically denotes the energy interaction (*E*_C_ or *E*_VDW_) arising from the continuity of the central segment of the A_6_H-33 model and must be added to the *E*_Center_ value for a direct comparison with that of the A_6_H-11 model.

After all these calculations, our results show that the Coulombic [van der Waals] interactions for the Ala-Ala pair, for the A_6_H-11 structure and central region of the A_6_H-33 nanomembrane, exhibit a difference of 0.1% [∼7%]. For the Ala-His interaction, which involves the polar head on the membrane surface, the observed values for the A_6_H-11 and central region of the A_6_H-33 show a more significant difference of the ∼8% [∼10%]. For the His-His interaction, these values are approximately −0.5% [∼6%]. In interactions with water, Ala-water results are more pronounced, indicating a percentage difference of ∼13% [~–376%]. It is worth noting the change in the van der Waals component, which shifts from repulsive when obtained with the A_6_H-11 structure to attractive when obtained with the central structure of A_6_H-33. Finally, the interaction between the surface of the structure and water molecules, governed by the His-water pair, highlights a variation of ∼7% [~–8%].

Thus, based on the obtained results, we can indicate that the central region of the A_6_H-33 simulated nanostructure appears to be more stable than the same portion of peptides simulated as a single structure, indicated as the A_6_H-11 membrane model. Almost all interaction energies are more intense in the macromembrane simulation compared to the simulation of the isolated central region. Therefore, the results regarding the energetic characteristics of the systems suggest that it is possible to obtain a more stable structure when simulating a membrane with larger dimensions. This may lead to configurations that generally describe more specific membrane characteristics that cannot be obtained with configurations simulated from a small region mimicking an infinite membrane through the application of PBCs. In particular, we can assess that the small region simulated with PBCs does not have access to membrane structures describing specific configurational domains that may be generated in the central configuration or near it, where there is no interference from PBCs. This may also be related to the possibility of large membranes having sufficient area for the formation of distinct domains but which obey the same main characteristics of the structures formed by self-assembly. Once again, we emphasize that such results do not invalidate simulations developed under a smaller surface aspect ratio, but we emphasize that even highly organized peptide structures can only have visible contributions when simulated in larger dimensions and that the percentages presented in this work can give a real idea the price that is paid when computational costs make analysis of a macro surface impractical.

### 3.2 Peptide Membrane’s Structure

In this session, we will carry out a structural evaluation of membranes depending on the size of the simulated surface. Some characteristics will be considered, such as the number of peptides per area, membrane thickness, and the organization of the β-sheets.

#### 3.2.1 Peptide Density per Unit Area

The nanostructures maintain a clear separation between hydrophilic and hydrophobic regions even after a MD production simulation. In this regard, a comprehensive quantitative analysis was conducted, considering a wide range of configurations in the models. The aim was to examine how peptides cluster in relation to the membrane area (peptides/nm^2^). The A_6_H-11 and A_6_H-13 models display higher compaction, indicating a tighter structure; we can also observe that the A_6_H structure, in general, exhibits a high clustering of peptides. This cluster, seemingly, shows a slight dependence on the simulated membrane area, indicating a variation of ∼2.5% when comparing the largest and smallest structures simulated. In comparison to peptide membranes formed by R_2_F_4_R_2_ monomers, the peptide/nm^2^ characteristic of A_6_H membrane is highly elevated. The values presented for the mentioned membrane formed by R_2_F_4_R_2_ are equal to 1.04–1.11 pep/nm^2^.^31^ This characteristic demonstrates that peptide membranes are very dependent on the peptide molecule in membrane formation and there may be distinct impacts from other peptide membranes when the surface area is increased. Therefore, the results presented here should be carefully evaluated and compared to other peptide structures. In general, we believe that our results can be useful in comparing structures formed mainly by alanines.

#### 3.2.2 Mass Density Profile in the *z* Direction

**Figure 4:**
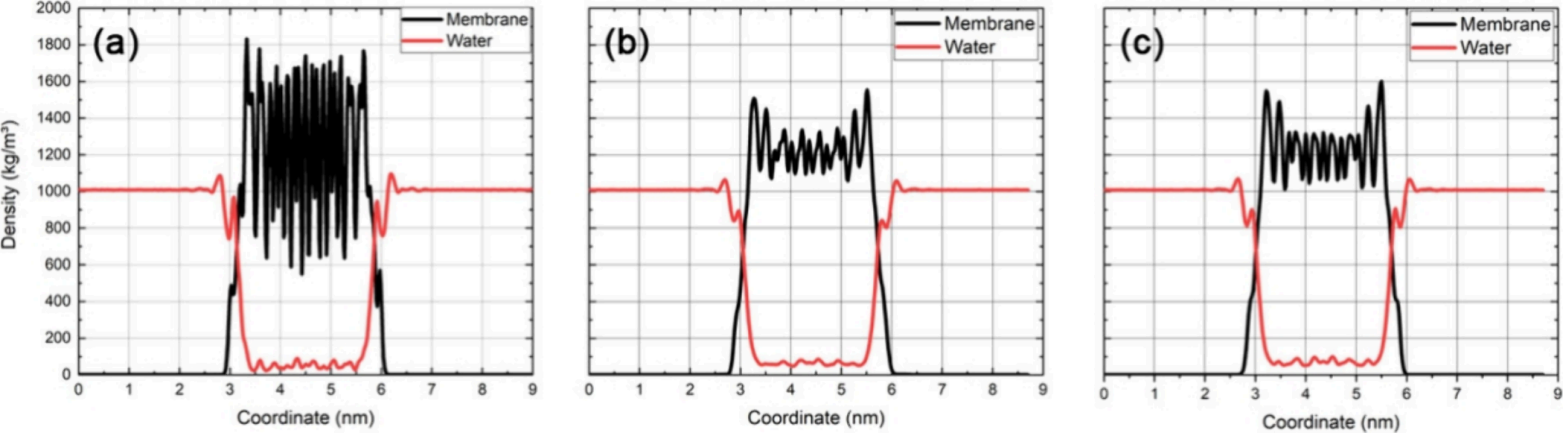
Average mass density profile in the *z* direction (in kg/m^3^) for peptides and water molecules of nanomembranes (a) A_6_H-11; (b) A_6_H-22; and (c) A_6_H-33 model. Figure S3 shows all mass density profile for all systems.

The mass density profile along the *z*-axis, perpendicular to the membrane surface, can provide insights into the hydrated region of the nanostructure and additional parameters for membrane thickness. This property can be determined by distance between the intersection of the mass density profile of peptides and the mass density profile of water molecules. It is also observed that certain models exhibit a low mass density of water molecules within the structure. The average thicknesses obtained for all models are shown in Table 2 and Figure 4 shown a difference in the silhouette of the mass density of peptides when comparing the models A_6_H-11, A_6_H-22, and A_6_H-33 in the region between 3 and 6 nm. As observed, the A_6_H-11 model exhibits a noisier distribution compared to the A_6_H-33 model, which displays a more organized structure. Comparing these values with the thickness obtained according to Castelletto et al.,^19^ we again observe a variation between 12 and 15%. This is expected, as this method of thickness calculation tends to overestimate results compared to values obtained with the SuAVE program (see Table 2). It is noteworthy that, in this regard, the results are minimally affected by the size of the membrane surface area, indicating that a model like A_6_H-11 can provide reliable estimates of the membrane structure.

**Table 2:** Thickness (in nm) of Each Peptide Membrane^a^

parameter	*e*_mass_	*e*_map_	Δ%
A_6_H-11	2.73	2.45 ± 1.08	10.3
A_6_H-12	2.70	2.48 ± 0.98	8.1
A_6_H-13	2.72	2.48 ± 0.99	8.8
A_6_H-22	2.71	2.43 ± 0.98	10.3
A_6_H-23	2.67	2.44 ± 1.07	8.6
A_6_H-33	2.70	2.43 ± 1.04	10.0

a Results obtained from the mass density profile (*e*_mass_) and from the surface map program built with the SuAVE program (*e*_map_). Δ% represents the percentage difference between the *e*_mass_ and *e*_map_ values.

#### 3.2.3 Membrane Thickness

An analysis of membrane thickness was conducted using the SuAVE program^47^ (see Table 2). This analysis involved measuring the N atom of the outermost histidine in the peptides. According to Castelletto et al.,^19^ the thickness of a dissolved A_6_H structure (in water and ZnCl_2_ solution) was measured to be 2.38 nm. However, our results showed variations between 2.1 and 4.2% when compared to the experimental value. Among these models, the A_6_H-22 and A_6_H-33 models exhibited the closest approximation to the experimentally obtained value. The results for this property measured with the SuAVE program^47^ assess the distance between two surfaces generated above and below the peptide membrane structure, indicating greater accuracy with an increase in the analyzed surface area. We believe that the results are moving toward convergence in our study; however, the choice of the reference atom may also suggest a new converged value for the property. Despite this theoretical–experimental difference, theoretical results obtained in other studies highlight values close to those obtained for the A_6_H-11 structure,^31^ emphasizing the need for additional analysis in structures with larger surface areas.

#### 3.2.4 Mass Density Profile in the *y* Direction

**Figure 5:**
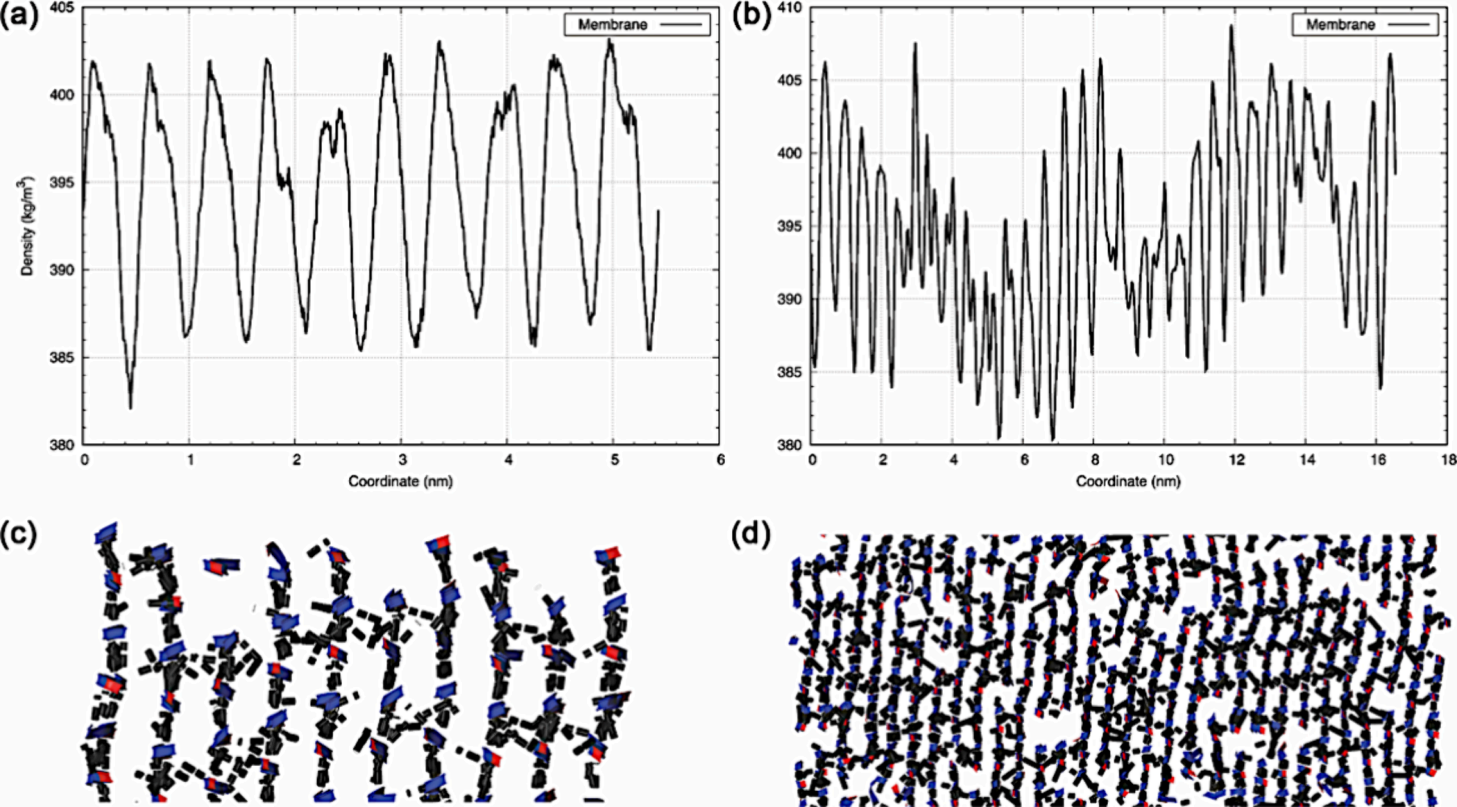
Average mass density profile (in kg/m^3^) in the *y* direction for peptide molecules. (a) A_6_H-11 model and (b) A_6_H-33 model. The figure also highlights, panels c and d, a frame with the distribution of peptides (in blue and red) in the *xy*-plane and hydrogen bonds (in black) that define the stacked β-sheets. It is worth noting that this highlighted configuration is one among 10^5^ configurations, and regions without hydrogen bonds in this snapshot may appear in others configuration, as this is a dynamic characteristic of the system.

To better observe the laminar separation of the β-sheet leaves of the peptides constituting the nanomembrane, we show in Figure 5 the mass density profile in the direction of the peptide sheets that make up the stacking of the β-sheets. As we can see, the A_6_H-11 structure exhibits a better organization of β-sheets, such that the peaks of the mass distribution projection along the *y*-axis highlight well-defined β-sheets. This is not the case for the A_6_H-33 structure, which shows a slight deformation in the distribution of stacked peptide β-sheets, appearing to have a slightly undulating distribution of these β-sheets. This is something that would be impossible to observe during the simulation of the smaller configurational structure (A_6_H-11) due to the reduced dimensions of the system. Despite this undulation, the characterization of the β-sheets is still evident. The average distances between the peaks for the presented systems are *P*_model-11_ = 0.54 nm and *P*_model-33_ = 0.61 nm, representing a percentage difference of ∼13% between the models. Experimental comparison can be made with data extracted from reference,^19^ which shows values that can reach up to 0.6 nm (depending on the percentage of water in solution) for the typical distance of β-sheets in A_6_H membranes. In this sense, our larger simulated system demonstrates more reliable results when compared to the experimental value. Figure 5 also provides a superficial view of the peptide positioning and their respective hydrogen bonds (HBs), highlighting the stacking of β-sheets for an MD-trajectory configuration. Note that the smaller structure does not exhibit undulations that can be observed in the larger structure. Although it may seem like a small difference, the interpretation that there may be a superordering in the structures is evident when analyzing only the A_6_H-11 membrane. However, a more realistic interpretation is only possible with the analysis of the A_6_H-33 membrane, which is 9 times larger than the previous structure. Finally, when dealing with finite size effects on membranes, capillary waves must be taken into account. These short wavelength fluctuations are absent in small-sized samples. Therefore, it is crucial to consider the effects of capillary waves when evaluating finite size effects and simulated membrane systems, highlighting the importance of conducting simulations with larger systems. As shown in the figure, there is a trend that emphasizes these capillary waves in the larger system (with peaks approximately 4 nm apart) compared to the smaller system.

### 3.3 Hydrogen Bond (HBs) Structure and Dynamics

#### 3.3.1 Hydrogen Bond Structure

**Figure 6:**
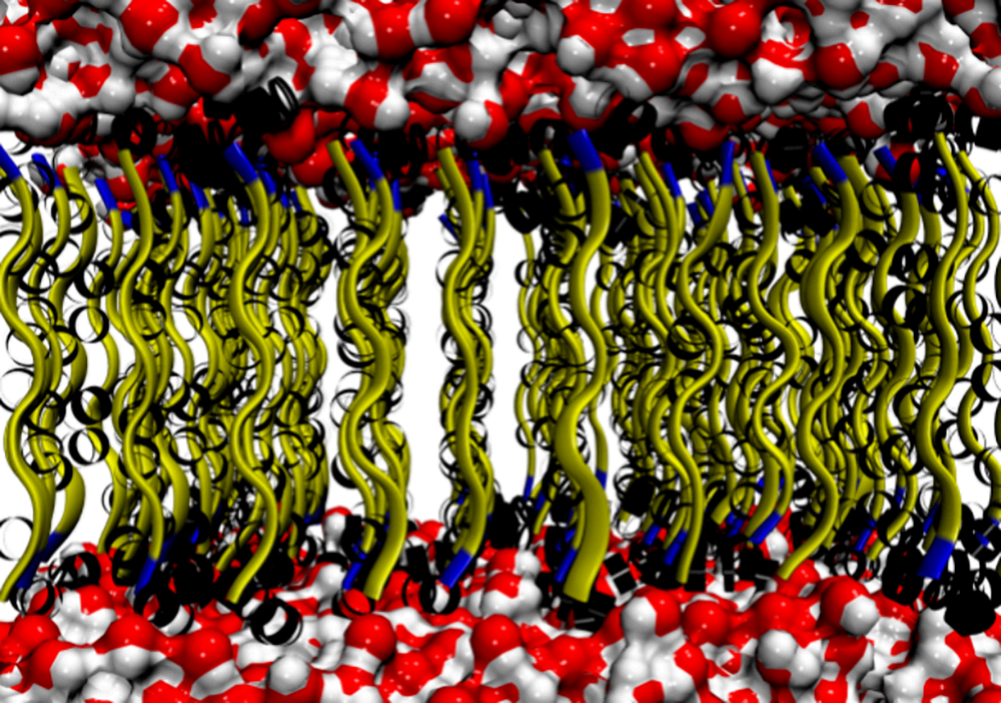
Orientation of HBs. In white and red, water molecules; in yellow, alanine; in blue, histidine; and in black, HBs regions.

Recent studies have highlighted the importance of HBs in maintaining membrane structures, influencing the arrangement of certain amino acids.^48,49^ HBs also play a role in permeability properties, as they can form between water molecules and polar groups in the membrane structure, allowing for hydration of these structures. These bonds can be formed and broken^1^ enabling changes and adaptation of the membrane structure. Figure 6 represents the HBs after 100 ns of MD simulation for model A_6_H-11. For this work, the HBs were calculated using the following parameters: *r* ≤0.35 nm and θ ≤30°.

Table S2 shows the average number of HBs per peptide (HBs/N) for all simulated nanostructures. For the Ala-Ala interaction, the results indicate some maintenance of the average HBs value with the increase in the surface area of the simulated nanostructure. Although this number is relatively stable, there is a difference in the volume of the simulated nanomembrane, causing the volumetric density of HBs between Ala-Ala to vary in each structure, ranging from 5.8 to 5.5 HB/nm^3^ within the nanomembrane. For the Ala-water interaction, the average number of HBs ranges from 2.67 to 2.89 HB/N, while for the Ala-His interaction, the average values remain close to 2.4 HB/N. Note that these two ratios (Ala-water and Ala-His) change relatively little. If we add the Ala-His count to the calculations of internal HBs in the membrane, the density of HBs/nm^3^ systematically decreases from 9.0 to 8.7 with the increase in the simulated membrane area. For the His-His interaction, the average values do not exceed 1 HB/N. Finally, for the interaction between the membrane surface and solvent, the His-water interaction, the results for HB/N are close to 5 HB/N, which demonstrates a small fluctuation in the solvent interaction in the boundary region of the membrane with the increase in the simulated area. All these results take into account the PBCs, which guarantees homogeneity of the average values. Additionally, it is important to highlight the significance of HBs in maintaining β-sheet structures, playing a crucial role in characterizing membrane structure, and serving as a key factor in keeping lamellae aligned. Structures with a higher average number of HBs may result in better self-assembly of the peptides, and we can observe that the A_6_H-11 model may be more favorable in this aspect. However, there is a need to evaluate how PBCs contribute to tethering, reducing the system’s freedom in self-organizing β-sheets and, consequently, hydrogen bonds (HBs).

One way to make this comparison is to assess the average number of HBs for the A_6_H-11 system and the central region of the simulated A_6_H-33 membrane system, considering the same criterion established in the comparative calculations of Coulombic and van der Waals energy conducted earlier. Thus, under these criteria, we observe that the average number of HBs shown a difference of less than 1 HB/N (the highest value found is 0.6 HB/N for the Ala-water interaction). However, the presence of 100 peptides in the analysis increases the average number of HBs in the simulated region by up to 60 HBs, which is a significant value for the simulated area. This implies that, statistically, the HBs between the Ala-Ala and His-His amino acids are affected, in terms of total number, by the existence of bonds established between opposite amino acids in the simulation box that are interacting by the conditions imposed in the PBCs. The interactions between Ala-water and Ala-His, for example, show an increase of 23 and 18%, respectively. Thus, we can observe that there is an impact on the average number of HBs depending on the area of the simulated structure for these structures based on alanine and histidine. In this way, simulations of reduced structures can lead to an interpretation that the structure is less correlated due to less interaction between amino acids and consequently indicate an interpretation that the structure is more malleable or less rigid.

For the average number of HBs, we can compare the results obtained for membrane systems composed mainly of alanines. Previous studies show that the average number of HBs in these systems is approximately 7.84, 6.92, and 4.50 HBs/peptide depending on the membrane structure formed by A_6_R;^50^ around 7.8 HBs/peptide in A_6_D-type structures; and about 8.2 HBs/peptide in membrane structures formed by A_6_K.^51^ As previously described, our estimate is that the average number of HBs between peptides increases by about 0.6 HBs/peptide. Therefore, the values described for the highlighted composite systems can be adjusted, estimating a result free from PBCs and reflecting an increase that may range from 7 to 13%.

#### 3.3.2 Energetic and Dynamic Analysis of HBs

Another crucial set of information derived from HBs calculations includes the HB-lifetime and Gibbs’ free energy value (Δ*G*) for HBs breaking, obtained through the Luzar–Chandler Theory.^42,43^ This involves computationally intensive calculations conducted on each of the 10^5^ frames extracted from the MD simulation trajectory. However, executing these calculations for the entire data set is impractical for large systems, such as the studied A_6_H-33 membrane. To acquire this vital information for these systems and discussion, we conducted the analysis using up to 10% of the frames of MD trajectories (equally spaced). To illustrate the convergence of #HBs/N, Δ*G* Gibbs’ energy, and HB-lifetime values, we will present the analyses for A_6_H-11 and complete A_6_ H-33 using only 50, 100, 200, 400, 800, 1250, 2500, 4000, 6600, 8250, or 10,000 configurations.

**Figure 7:**
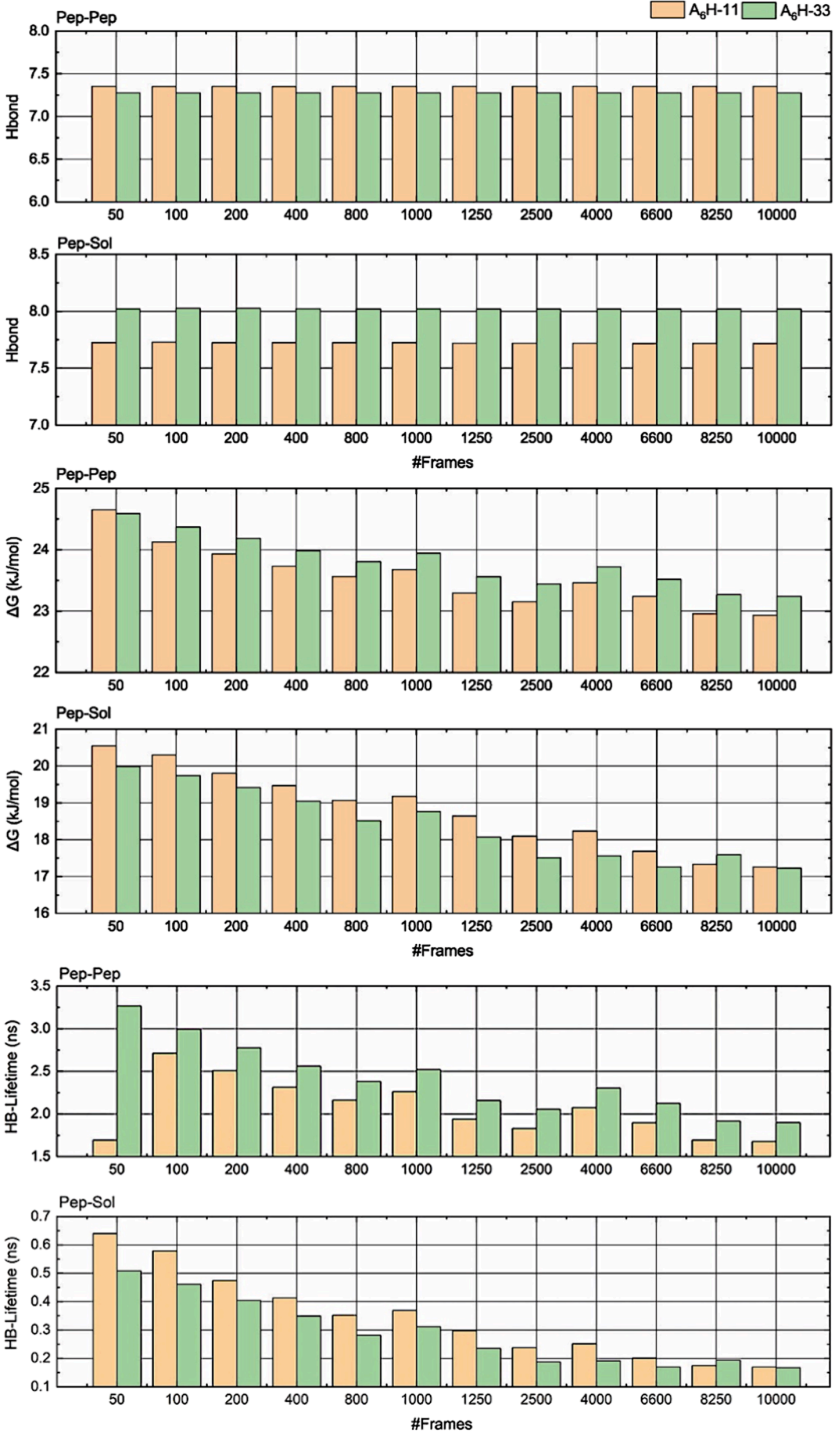
HB (#HBs/N); Δ*G* (in kJ/mol); and HB-lifetime (in ns) for the A_6_H-11 and A_6_H-33 system that demonstrate convergence of statistics analyzes for up to 10^4^ frames of the MD-trajectory.

For the average number of HBs/N in A_6_H-11 and A_6_H-33 systems, the results show (see Figure 7) that the average value is practically constant, regardless of the quantity of frames selected from the MD-trajectory. Regarding the variation in Gibbs’ free energy (Δ*G*), we observe that the peptide–peptide (Pep-Pep) interaction does not change significantly, ranging between 23 and 25 kJ/mol for all subensembles selected for statistical analysis. However, for the peptide–water (Pep-Sol) interaction, the averages values decrease from ∼21 to ∼17 kJ/mol, converging together to the same estimate value, emphasizing that the differences for Δ*G*_Pep-water_ are not significant when comparing results obtained to A_6_H-11 and A_6_H-33 membrane using 10^4^ configurations. Finally, the HB-lifetime shows a variation of 1.5–3.2 ns when obtained for the peptide–peptide pair (using only 50configurations) and a variation of 1.6–1.9 ns (using 104 configurations of MD-trajectory). It can beobserved that, in this case, there is a convergence of the property with around 6000 configurationsselected for statistical analysis. For the peptide–water interaction, the decrease in HB-lifetime values is significant with the increase in the selected configurations and the property converges to around 0.15 ns in both subsystems analyzed. This result is essential, as it enables an energetic and dynamic analysis of HBs with a relatively small computational cost for very large systems. Therefore, the following analysis takes into account data extracted using 10^4^ configurations that, apparently, generate practically converged values for the studied membrane systems.

Thus, based on this information, we conducted new calculations for simulated systems using the same parameters, with 10% of the frames obtained from the MD-trajectory (only 10^4^ configurations). The values for the A_6_H-11 systems were obtained using 100% of the trajectory frames for comparison. Table 3 presents the results obtained from this analysis. The results for number of HBs show a consistent average value for the number of HBs/N, regardless of the size of the studied system, indicating that, when evaluating the complete systems under similar PBC, there is no significant variation in the average number of HBs. As an example, we highlight that for the smallest [largest] system, the average number of HBs/N (Pep-Pep) converges to approximately 7.36 [7.28] HBs/N, and the average number of HBs/N (Pep-Sol) converges to a value of about 7.71 [8.02] HBs/N. Therefore, as the total number of HBs/N is not significantly affected, we can conduct a statistical analysis on such parameters to obtain the properties of Gibbs’ free energy (Δ*G*) and HB-lifetime, as outlined below. The value of the Gibbs free energy, for breaking HBs, converges to values that maintain a difference between the results obtained below 1 kJ/mol. However, the HB-lifetime between peptides varies when obtained for the smallest and largest membranar systems. The findings reveal that the HB-interaction persists for around 1.6 ns in the A_6_H-11 system, whereas in the A_6_H-33 system, it endures for about 1.9 ns. Peptide–water interactions oscillate between 0.1 and 0.2 ns, a typical result found in other studies such as de Almeida et al.^49^ and findings by van der Spoel.^44^Table 3 provides a detailed breakdown of these results, comparing them with those obtained for the center of the A_6_H-33 structure, elucidating the influence of PBCs on this statistical analysis.

**Table 3:** Values Obtained for HBs (in #HBs/N); Δ*G* (in kJ/mol); and HB-lifetime (in ns) from the Luzar-Chandler Theory^43^ Using Only 10% of MD-Trajectory^a^

parameter	A_6_H-11	A_6_H-12	A_6_H-13	A_6_H-22	A_6_H-23	A_6_H-33	center of A_6_H-33
# HB/N							
Pep-Pep	7.35	7.46	7.49	7.30	7.35	7.28	7.96
Pep-Sol	7.71	7.60	7.51	7.94	7.81	8.02	8.58
Δ*G*							
Pep-Pep	22.82	23.30	22.59	23.04	23.19	23.24	23.62
Pep-Sol	16.59	16.93	17.41	16.22	16.93	17.22	17.04
HB-lifetime							
Pep-Pep	1.6	1.9	1.5	1.8	1.9	1.9	2.2
Pep-Sol	0.1	0.1	0.2	0.1	0.1	0.2	0.2

a Only A_6_H-11 use all frames of the MD-trajectory for statistical analyses and comparisons.

Finally, it is important to highlight the differences found when comparing the smallest simulated model (A_6_H-11) with data extracted from the central region of the simulated membrane in the A_6_H-33 model. Table 3 also illustrates such comparisons, and as can be observed, the central model, free from the constraints imposed by PBCs, exhibits higher average values for the properties (compared to all other models). For the average number of HBs per peptide in the simulated region (HBs/N), we can calculate an increase of about 0.6 HBs/N, representing a total difference of about 60 HBs in the simulated area. This implies an increase in the density of HBs per unit area of material (approximately 2 HBs/nm^2^). Thus, the model with the smallest simulated area (A_6_H-11) underestimates the results, while the model with the largest simulated area (A_6_H-33) predicts results closer to what can be expected, when PBCs are not effectively considered in a region specific analysis. These results are of enormous theoretical value as they demonstrate the need to evaluate the impact of the size of the simulated structure, especially for results involving interactions between the peptide nanomembrane and water such as the values of the energetic barriers and the lifetime of these interactions. Finally, for this last property, we can observe a difference of up to 0.6 ns between the peptide–peptide interaction obtained by analyzing the entire A_6_H-11 model and the central region of the A_6_H-33 model. This is a surprising result that demonstrates that the A_6_H-based peptide structure can be considered significantly more robust/structured than predicted by simulating a small region of the membrane treated as infinitely large with the application of PBCs.

#### 3.3.3 Ramachandran Plots and Einstein’s Diffusion Coefficient

**Figure 8:**
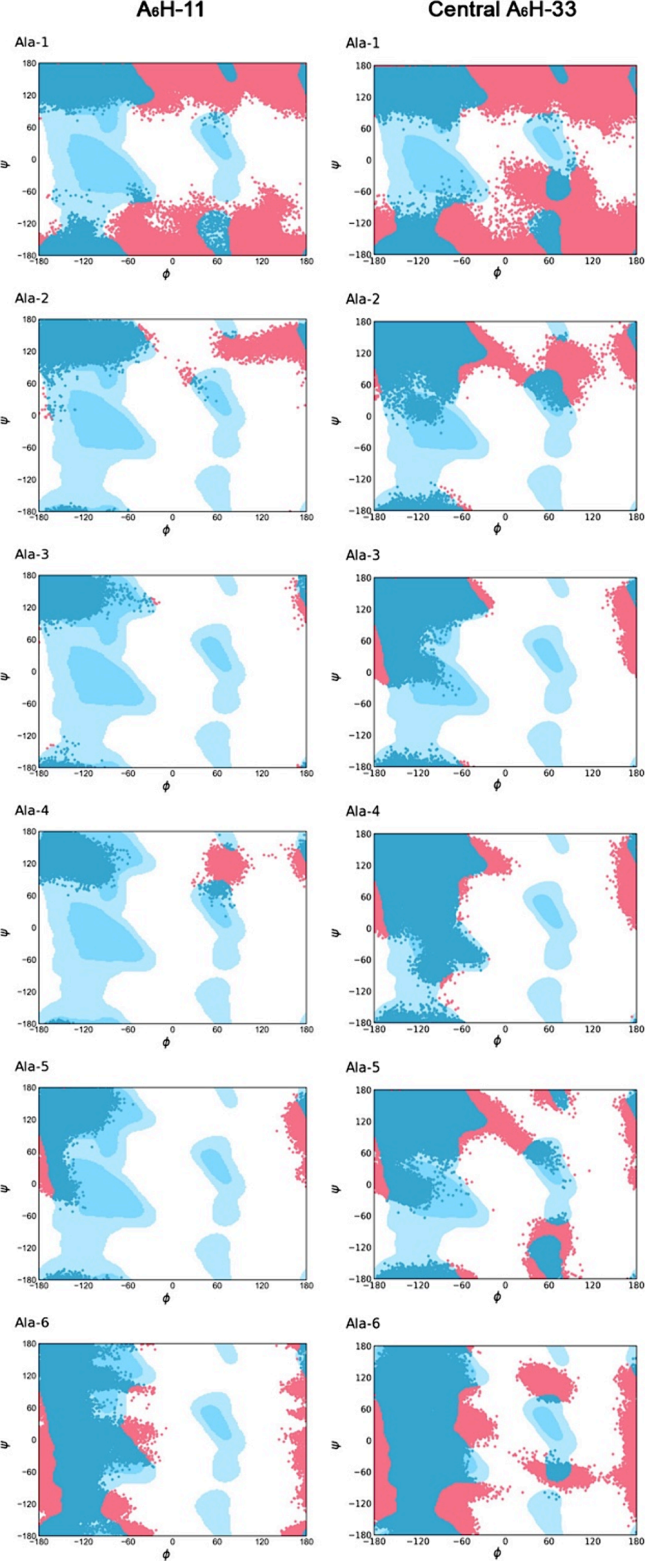
Ramachandran plots for the alanine residues comprising (left) model 1 × 1 and (right) the central part of model 3 × 3. The red regions indicate the ψ and φ angle distribution for each configuration of the MD trajectories. The blue regions indicate the most common angles for alanine residue.

An important comparison concerns the mobility of peptides when comparing models A_6_H-11 and the central region of model A_6_H-33. Figure 8 displays Ramachandran plots highlighting the behavior of the ψ and φ angles for each alanine residue within the core of the peptide membrane. It can be observed that the central model exhibits greater mobility of psi/phi angles across all six alanine residues compared to the smaller model (A_6_H-11), as evidenced by more filled plots in Figure 8 (right). This increased mobility in the central region indicates that PBCs make the system stiffer and restrict peptide movement within the membrane structure. However, it is worth noting that this increased mobility does not lead to a disruption of the proposed membrane structure. Additionally, lateral Einstein’s diffusion coefficient (MSD) was calculated for these same peptides analyzed in the Ramachandran plots. Our results showed lateral MSD values of 9.6 × 10^–7^ cm^2^/s for collective displacement of peptides in the smaller structure (A_6_H-11) and values of 9.0 × 10^–8^ cm^2^/s for the center of structure A_6_H-33. This set of results indicates that, in the central structure, free from PBCs, the peptide network exhibits more unrestricted vibration, favoring a grated distribution of points in the Ramachandran plots but with lower lateral mobility, which is expected for small membranes when simulated within the formation plane inside the simulation box. These findings are consistent with previous research and underscore the importance of adequate membrane modeling for this type of organic material.

## 4 Conclusions

The MD simulations conducted in this study provide a systematic and detailed analysis of molecular interactions and structural properties of peptide nanomembranes, especially those formed by alanine (A) blocks and histidine (H) polar heads, A_6_H peptide. The results indicate that, for electric interactions, there are no significant variations among models of different sizes that were simulated, suggesting that the increase in membrane area does not significantly impact these interactions. However, van der Waals interactions exhibit some variations, particularly in the interaction between alanine and water, indicating a more pronounced influence of membrane size in this context, highlighting regions where there is increased water entry into the nanomembrane model. Structural analyses reveal differences in peptide densities per simulated material area, emphasizing greater compression in A_6_H-11 and A_6_H-13 membranes, indicating that membrane size influences the organization and density of peptides. Additionally, membrane thickness analysis shows convergence to more accurate values as the analyzed area size increases, suggesting that the choice of analysis region size can impact the interpretation of structural properties and the arrangement of stacked peptide β-sheets in nanomaterial formation.

Detailed analysis of HBs reveals subtle variations in Ala-Water and Ala-His interactions with increasing membrane area. Furthermore, the comparison between the A_6_H-11 model and the central region of the A_6_H-33 model, which have the same number of simulated peptides but with and without constraints imposed by PBCs, respectively, highlights the impact of PBCs on interpreting the average and energetic properties of HBs. There are significant differences in HBs characteristics and structural stability, demonstrating a significant difference in the lifetime of these interactions, which can affect the interpretation and assessment of material stability and robustness, directly impacting its application. Thus, these energetic and dynamic analyses of HBs emphasize the importance of considering membrane size in interpreting results. The convergence of properties observed with the increase in analyzed area, along with the analysis of a PBC-free region, suggests the need for more comprehensive approaches to simulating peptide nanomembranes, especially when seeking to understand critical properties such as structural stability and the dynamics of molecular interactions in self-organizing systems. The analysis of Ramachandran plots and lateral Einstein’s Diffusion Coefficient has provided valuable insights into the mobility and structural behavior of peptides in the studied systems. With these properties, it can be observed that the smaller model exhibits less flexibility than predicted for the central model when simulated free from PBCs. These findings, which highlight the influence of PBCs on the properties of self-organizing systems, can be more pronounced in organizational properties such as the alignment of β-sheets and consequently the statistics of HBs. Thus, the results observed in this study also corroborate findings obtained for systems with higher mobility or disorder in molecular aggregation, such as lipid membranes. For these types of structures, it has also been demonstrated that PBCs produce results dependent on the size of the simulated systems, as described by Klauda and collaborators.^29^ Thus, our study compares a conventional-sized peptide system with a larger system, investigating the impact of PBCs, which are less pronounced in the central part of the membranar structure in the larger box simulation. The results reveal significant differences in the average number and duration of hydrogen bonds between the analyzed systems, and the robust methodology (including convergence criteria and selection of simulated structure dimensions) ensures the validity of the results as a reference for simulations of A_6_H-type peptide membrane structures, where interactions in the hydrophobic region are predominantly among alanines.

## Supporting Information Available

Presents graphs highlighting an energy analysis and system energy convergence (Figures S1 and S2), tables with specific values used to construct the manuscript graphs (Tables S1 and S2), and detailed results for the mass density profile of all studied systems (Figure S3) (PDF)

## Notes

Any correspondence concerning this work can be forwarded Guilherme Colherinhas (gcolherinhas@ufg.br) or Karinna Mendanha (karinnamendanha@discente.ufg.br).
