# Supplementary material for: Exploring How System Dimensions and Periodic Boundary Conditions Influence the Molecular Dynamics Simulation of A6H Peptide Self-Assembly Nanostructures
Source: J Phys Chem B. 2024 Jul 9;128(28):6853–65. doi: 10.1021/acs.jpcb.4c03043 (PMC11264264; doi:10.1021/acs.jpcb.4c03043)
Supplement: Supplementary file 1 — jp4c03043_si_001.pdf [file jp4c03043_si_001.pdf]

SUPPORT INFORMATION MATERIAL

Exploring How System Dimensions and Periodic Boundary Conditions Influence the Molecular Dynamics Simulation of A_6H Peptide Self-Assembly Nanostructures

Karinna Mendanha[a] and Guilherme Colherinhas[a*]

[a] Instituto de Física. Universidade Federal de Goiás. 74690-900. Goiânia. GO. Brazil.

** Corresponding author. E-mail address: gcolherinhas@ufg.br*

ARTICLE INFO

Keywords:
Molecular Dynamics;
Peptide;
H-bond;
HB-Lifetime;
Periodic Boundary Conditions.

ABSTRACT

This work presents the effects of periodic boundary conditions (PBC) on energetic/structural properties and hydrogen bond dynamics (HB) obtained from molecular dynamics (MD) simulations of large and small peptide membranes formed by alanine and histidine. Our results show that analyses conducted on small membrane structures may lead to non-converged values and may obscure structural properties that are only observed in a large simulation area of the peptide self-assembly nanomaterial. Regarding hydrogen bonds. a property that permeates membranes of this kind. our results indicate a significant increase in the lifetime of these interactions among peptides. reaching values ~19% higher when observed in structures with dimensions in a 1:9 ratio.

Graphical Abstract

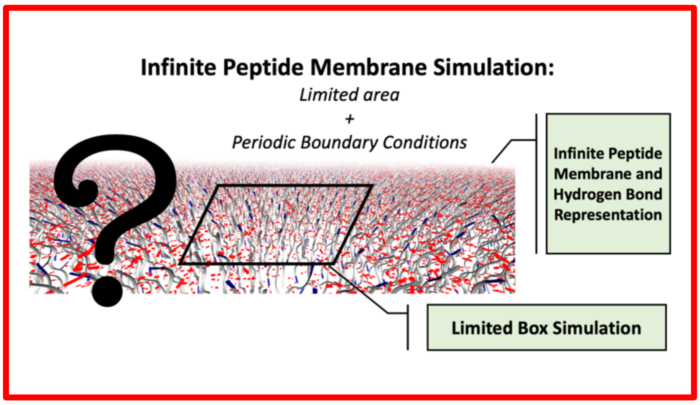

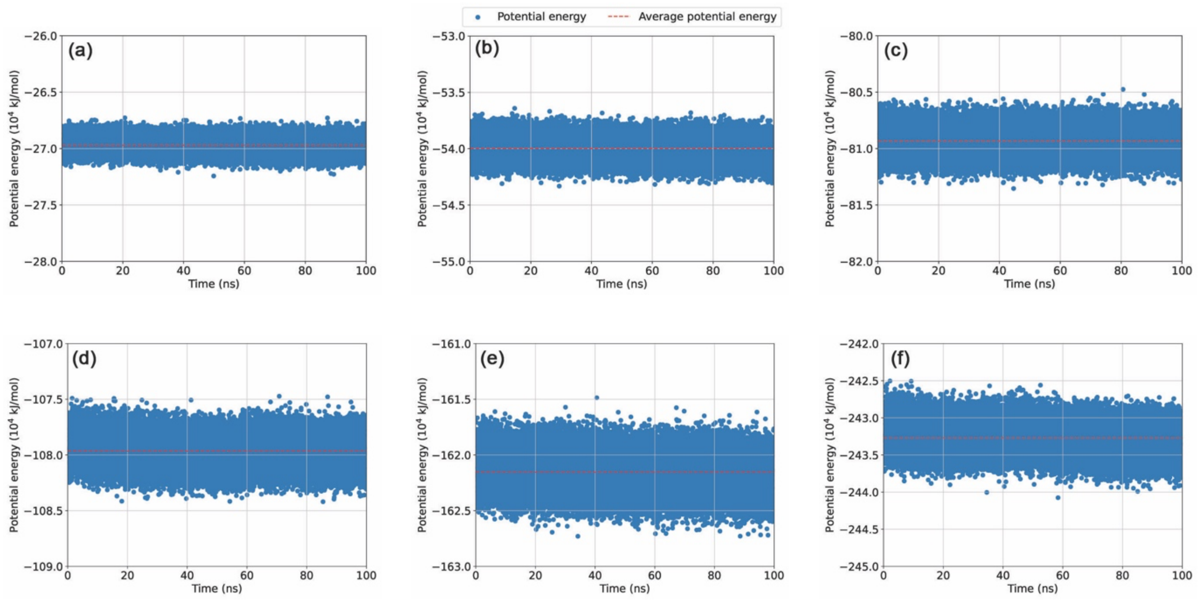

Figure S1: *Potential Energy for all system obtained in production steep. (a) A_6H-11, (b) A_6H-12, (c) A_6H-13, (d) A_6H-22, (e) A_6H-23, and (f) A_6H-33*

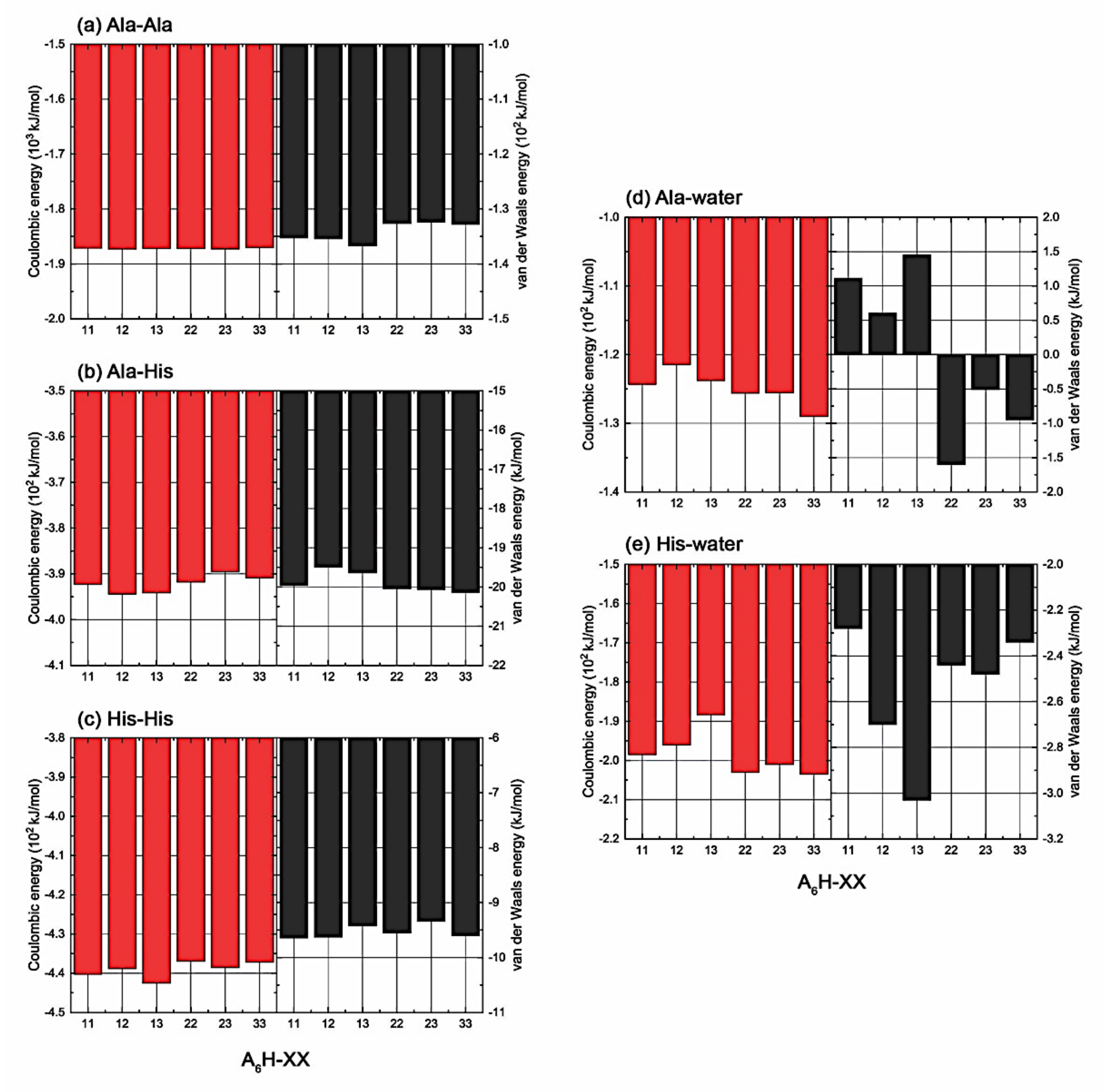

Figure S2: *Average Coulomb (red) and van der Waals (black) energy for interactions between residues (a,b, and c) and between residues and water (d and e),in kJ/mol.N, for the all A_6H-XX models. Results shown per peptide unit in nanostructures (N).*

Table S1: *Average Coulombic and vdw energy for interactions between residues and between residues an solvent (in kJ/mol) for the 6 models studied. Results shown per peptide unit.*

Interaction	A_6H-11	A_6H-12	A_6H-13	A_6H-22	A_6H-23	A_6H-33
Coulombic energy						
**Ala-Ala**	-1871.36 ± 2.59	-1873.04 ± 1.61	-1871.91 ± 1.25	-1872.22 ± 1.31	-1872.83 ± 0.91	-1870.44 ± 0.83
**Ala-Sol**	-124.31 ± 3.47	-121.45 ± 2.14	-123.78 ± 1.64	-125.62 ± 2.13	-125.58 ± 1.37	-129.03 ± 1.42
**Ala-His**	-392.26 ± 2.43	-394.39 ± 1.86	-394.15 ± 1.60	-391.78 ± 1.39	-389.54 ± 1.24	-390.91 ± 1.14
**His-His**	-440.24 ± 1.81	-438.83 ± 1.34	-442.54 ± 1.03	-436.94 ± 0.91	-438.53 ± 0.89	-437.20 ± 0.60
**His-Sol**	-198.48 ± 3.65	-196.06 ± 2.62	-188.32 ± 2.19	-203.00 ± 2.00	-201.05 ± 1.67	-203.47 ± 1.58
**Pep-Pep**	-2703.88 ± 3.34	-2706.29 ± 3.51	-2708.53 ± 1.94	-2701.00 ± 1.88	-2701.20 ± 1.39	-2699.04 ± 1.45
**Pep-water**	-322.79 ± 4.95	-317.52 ± 2.34	-336.07 ± 3.01	-328.63 ± 3.14	-326.63 ± 2.36	-332.51 ± 2.47
VdW energy						
**Ala-Ala**	-135.25 ± 0.96	-135.38 ± 0.62	-136.68 ± 0.51	-132.65 ± 0.57	-132.35 ± 0.40	-132.76 ± 0.33
**Ala-water**	1.11 ± 1.19	0.60 ± 0.73	1.45 ± 0.59	-1.60 ± 0.54	-0.51 ± 0.45	-0.95 ± 0.38
**Ala-His**	-19.96 ± 1.00	-19.49 ± 0.71	-19.64 ± 0.60	-20.04 ± 0.51	-20.06 ± 0.41	-20.14 ± 0.34
**His-His**	-9.64 ± 0.54	-9.63 ± 0.38	-9.42 ± 0.33	-9.55 ± 0.26	-9.34 ± 0.22	-9.60 ± 0.18
**His-water**	-2.28 ± 1.16	-2.70 ± 0.81	-3.03 ± 0.66	-2.44 ± 0.59	-2.48 ± 0.48	-2.34 ± 0.40
**Pep-Pep**	-164.86 ± 1.47	-164.49 ± 1.00	-165.71 ± 0.81	-162.19 ± 0.60	-161.67 ± 0.61	-162.38 ± 0.51
**Pep-water**	-1.17 ± 1.70	-2.10 ± 1.10	-1.58 ± 0.88	-4.04 ± 0.80	-3.00 ± 0.66	-3.29 ± 0.55

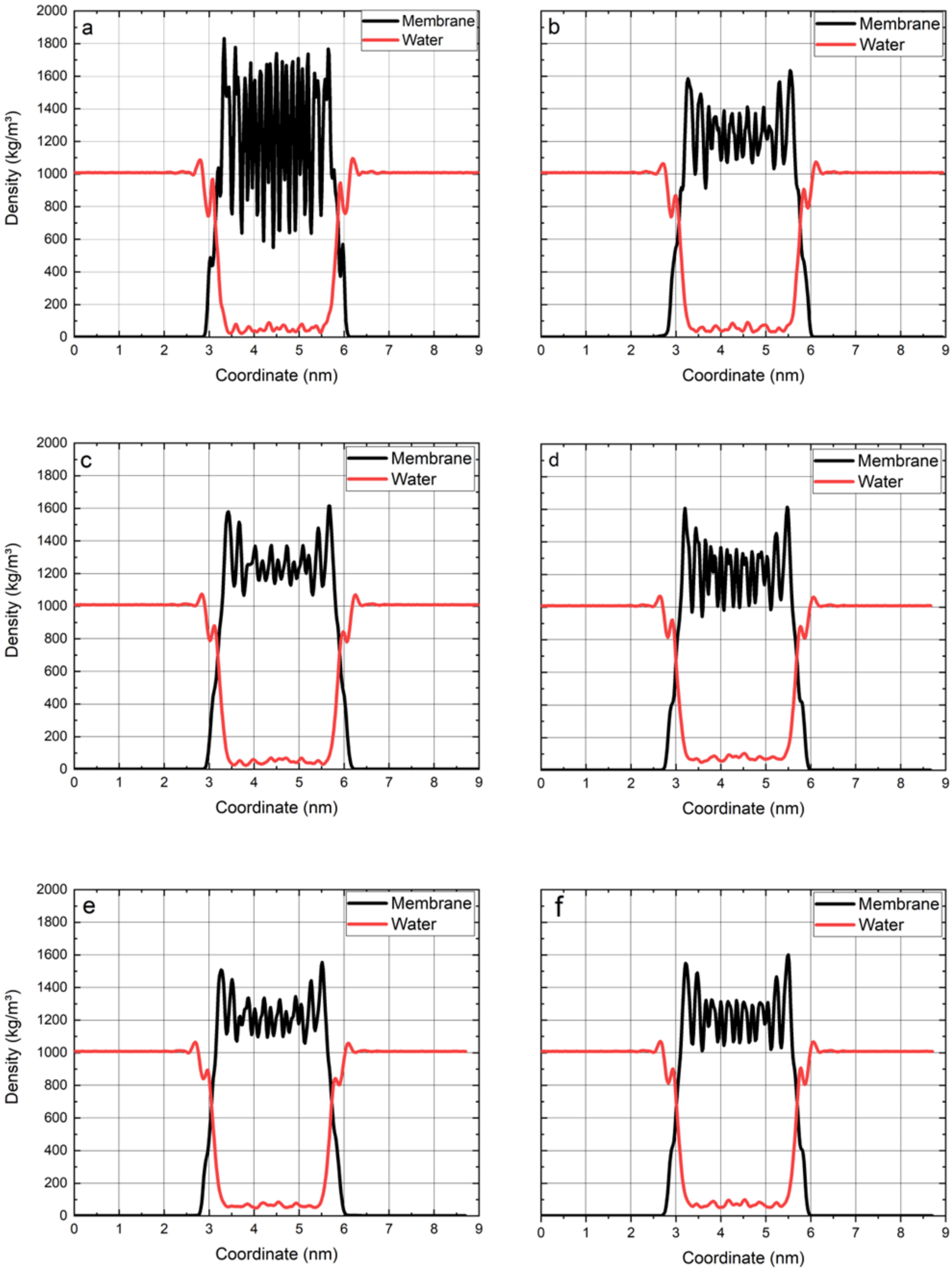

Figure S3: *Average mass density profile in z direction (in kg/m³) for peptides and water molecules of nanomembranes (a) A_6H-11; (b) A_6H-12; (c) A_6H-13; (d) A_6H-21; (e) A_6H-22; and (f) A_6H-33 model.*

Table S2: *The average number of hydrogen bonds per peptide unit.*

Interaction	***A_6H-11***	***A_6H-12***	***A_6H-13***	***A_6H-22***	***A_6H-23***	***A_6H-33***
Ala-Ala	4.24	4.31	4.31	4.21	4.24	4.19
Ala-water	2.67	2.61	2.66	2.82	2.74	2.89
Ala-His	2.41	2.48	2.43	2.41	2.40	2.41
His-His	0.74	0.68	0.75	0.67	0.70	0.67
His-water	5.03	4.99	4.85	5.11	5.07	5.12
Pep-Pep	7.35	7.46	7.49	7.30	7.35	7.28
Pep-water	7.71	7.60	7.51	7.94	7.81	8.02
